# Opioid-Sparing Effect of an Ultrasound-Guided Transversus Abdominis Plane Block Added to a Multimodal Analgesic Protocol in Dogs with Acute Pancreatitis: A Matched-Control Pilot Study

**DOI:** 10.3390/ani16213523

**Published:** 2026-11-07

**Authors:** Alba Díaz-González, Eva M. Pérez-Merino, José Ignacio Cristóbal-Verdejo, Carlos Martínez-Zafra, Lorena Espadas-González

**Affiliations:** Department of Animal Medicine, Hospital Clínico Veterinario, Facultad de Veterinaria, University of Extremadura, Avenida de la Universidad s/n, 10003 Cáceres, Spain; albadiazglez@gmail.com (A.D.-G.); jicristob@unex.es (J.I.C.-V.); carlosmarzafra1d@gmail.com (C.M.-Z.); lorenaeg@unex.es (L.E.-G.)

**Keywords:** acute pancreatitis, transversus abdominis plane block, regional anesthesia, opioid-sparing, pain management, dog

## Abstract

Acute pancreatitis in dogs causes severe abdominal pain, typically managed with systemic opioids, which can impair gastrointestinal motility and delay recovery. This prospective clinical communication evaluated the opioid-sparing effect and clinical outcomes of an ultrasound-guided transversus abdominis plane block added to a multimodal analgesic protocol in dogs with acute pancreatitis. Twelve dogs were allocated into a locoregional block group (*n* = 6) and a control group managed with opioids (*n* = 6), matched on five severity scores recorded at admission. The primary outcome was opioid consumption during the first 24 h after enrollment; secondary outcomes were pain during the same period, time to first voluntary food intake, hospitalization length, and inflammatory response. The block group received significantly fewer methadone doses (median 0 vs. 4.5; *p* = 0.015) and five of six dogs required no methadone beyond the enrollment dose. Cumulative pain scores during the first 24 h were also significantly lower in the block group (*p* = 0.037). The time to first voluntary food intake was shorter in the block group (12 vs. 48 h; *p* = 0.02). No significant differences were found in hospitalization length or survival. Adding a transversus abdominis plane block to a multimodal protocol was associated with a marked opioid-sparing effect and promoted earlier voluntary food intake, although these preliminary findings require confirmation in larger studies.

## Simple Summary

Dogs suffering from acute pancreatitis experience severe abdominal pain, which is typically managed with medications like opioids. While effective, these drugs often cause unwanted side effects, such as slowing down the digestive system, which can delay the patient’s recovery. This study investigated whether administration of a local anesthetic into the abdominal wall musculature could effectively relieve pain and reduce the reliance on systemic opioid administration. We compared six dogs treated with this locoregional block against six dogs managed with opioids, all still painful after a first non-opioid analgesic and all on the same baseline medication. Dogs receiving the local block required significantly fewer opioid doses and showed lower pain scores. Notably, five of the six dogs in this group did not need any additional systemic opioids during their first 24 h in the hospital. Furthermore, these patients regained their appetite and started eating independently earlier than those receiving standard care. In conclusion, incorporating this localized anesthetic technique into a multimodal pain management strategy may be a valuable approach for dogs with acute pancreatitis, as it reduced the need for systemic opioids, and was associated with an earlier return to eating, which may improve animal welfare. Being a small preliminary study, these results need confirmation in larger trials.

## 1. Introduction

In dogs, acute pancreatitis (AP) is an acute inflammatory condition of the exocrine pancreas characterized by pancreatic edema, acinar cell injury, necrosis, and infiltration of inflammatory cells within the exocrine parenchyma, leading to clinical signs such as vomiting, diarrhea, anorexia, and severe abdominal pain [1,2,3,4]. Hospital management protocols include intravenous fluid therapy, nutritional support and supportive care, with pain control being an essential component of treatment. Currently, analgesic management relies primarily on systemic opioids [5]. Although effective, opioids are associated with several adverse effects, including decreased gastrointestinal motility, nausea, sedation, and dysphoria. These effects may delay the resumption of voluntary food intake, potentially prolonging hospitalization and increasing the risk of complications [6,7]. In human medicine, increased opioid consumption has been linked to prolonged hospitalization and poorer outcomes [8,9]. Consequently, there is growing interest in opioid-sparing strategies, including regional anesthesia techniques such as the transversus abdominis plane (TAP) block [10,11].

This regional anesthetic technique provides analgesia to the ventral and lateral abdominal wall [12] by depositing local anesthetic between the transversus abdominis and the obliquus internus abdominis muscles, blocking the ventral branches of thoracolumbar spinal nerves [13,14]. In human medicine, the TAP block is routinely used for abdominal surgery [15,16,17] and, in patients with AP, it has relieved pain and reduced both opioid consumption and hospitalization time [18,19]. In veterinary medicine, the TAP block has been primarily described in surgical procedures such as ovariectomy and mastectomy [20,21,22,23], while evidence in AP remains scarce. To date, a single case series has described its use in three dogs—two of which developed pancreatitis following laparotomy (for pancreatic mass removal and foreign body extraction)—utilizing continuous TAP blocks via surgically placed catheters [24]. However, no studies have yet evaluated the efficacy of a single-injection, ultrasound-guided TAP block specifically for reducing opioid requirements in dogs with AP managed medically, without concurrent abdominal surgery.

Therefore, this prospective, non-randomized, matched-controlled study aimed to investigate the clinical utility of an ultrasound-guided TAP block in dogs with acute abdominal pain secondary to AP. Specifically, we evaluated whether incorporation of this locoregional technique into a multimodal analgesic protocol could reduce systemic opioid requirements while maintaining adequate control of abdominal pain, and improve markers of early clinical recovery. We hypothesized that dogs receiving a TAP block would require less opioid administration and achieve a faster clinical recovery than dogs managed with the same protocol without the locoregional block.

## 2. Materials and Methods

### 2.1. Study Design and Patient Screening

This prospective, non-randomized, controlled clinical study was conducted at the Veterinary Teaching Hospital of the University of Extremadura (VTH-UEx) between September 2025 and January 2026. The study was approved by the University of Extremadura (UEx) Animal Care and Use Committee (register number 15/2025), and written informed consent was obtained from all owners, including specific consent for the TAP block when applicable.

Client-owned dogs admitted with a clinical suspicion of AP were screened for inclusion. Inclusion required acute abdominal pain accompanied by at least one additional compatible clinical sign (vomiting, diarrhea, nausea, anorexia, or lethargy). The diagnosis was confirmed at admission by a serum lipase activity > 160 U/L and at least two ultrasonographic findings consistent with AP (pancreatic enlargement, hypoechoic parenchyma, hyperechoic peripancreatic fat, or peripancreatic fluid accumulation) [25,26,27].

Dogs were excluded if they had received analgesics, including owner-administered non-steroidal anti-inflammatory drugs, before admission, either as long-term treatment or during the current episode, or if they had a concurrent gastrointestinal disease or any other painful condition that could interfere with pain assessment.

### 2.2. Baseline Assessment and Standardized Initial Care

Upon admission, comprehensive baseline data were recorded for each eligible patient, including signalment (breed, age, sex, body weight, and body condition score). Laboratory assessment included a complete blood count (ProCyte DX; IDEXX Laboratories, Westbrook, ME, USA) and a serum biochemical profile (Spin200E; Spinreact, Barcelona, Spain). Serum lipase activity and C-reactive protein (CRP) were measured using an automated dry chemistry analyzer (Element CM XT; scil animal care company GmbH, Hessen, Germany). To ensure consistency and minimize inter-observer variability, all ultrasound evaluations were performed by a single experienced sonographer.

Disease severity at admission was assessed with five scoring systems: the Canine Acute Pancreatitis Clinical Severity Index (CAPCSI) [1], the Acute Patient Physiologic and Laboratory Evaluation (APPLE) [28], the Modified Canine Activity Index (MCAI) [29], the clinical severity score proposed by Su et al. [30] and the Ultrasound Pancreas-specific Scoring System (UPASS) [25].

All dogs received the same initial stabilization intravenous fluid therapy with lactated Ringer’s solution (Lactato-Ringer; B. Braun VetCare S.A., Rubí, Spain), supplemented with potassium (Cloruro de Potasio Braun Uso Veterinario 149 mg/mL, B. Braun VetCare S.A., Rubí, Spain) and/or glucose (Glucosada Grifols 50%, Laboratorios Grifols S.A., Parets del Vallès, Spain) according to individual analytical and electrolyte requirements and dehydration status; maropitant (Prevomax 10 mg/mL; Dechra Veterinary Products SLU, Barcelona, Spain; 1 mg/kg IV q24h) and metamizole (Metamizol Normon 2 g/5 mL; Laboratorios Normon S.A., Madrid, Spain; 15 mg/kg IV q8h) as baseline analgesia. In dogs with persistent emesis, metoclopramide (Vomend 5 mg/mL; Dechra Veterinary Products SLU, Barcelona, Spain) was added as second-line antiemetic (constant rate infusion [CRI], 1–2 mg/kg/day). Water and a gastrointestinal diet (Veterinary Gastrointestinal; Royal Canin, Aimargues, France) were available ad libitum, and fresh palatable food was offered every 4–8 h to record the time to first voluntary intake.

### 2.3. Enrollment and Group Allocation

Pain was assessed using the short form of the Glasgow Composite Measure Pain Scale (CMPS-SF) [31] one hour after initial stabilization and administration of the first dose of metamizole. Dogs scoring ≥ 6/24, the validated analgesic intervention level of the scale [31], were enrolled. The time of this assessment was defined as T0, and all subsequent time points are expressed relative to it. The study population therefore consisted of dogs with AP whose pain persisted despite baseline non-opioid analgesia. Dogs scoring < 6/24 continued on the baseline protocol and were not enrolled in the study.

Group allocation was not randomized. Enrolled dogs were allocated to the TAP group when the anesthesiologist who performed all blocks was available and the owner consented to the procedure; the remaining enrolled dogs, recruited concurrently over the same period, formed the pool of potential controls.

**Figure 1:**
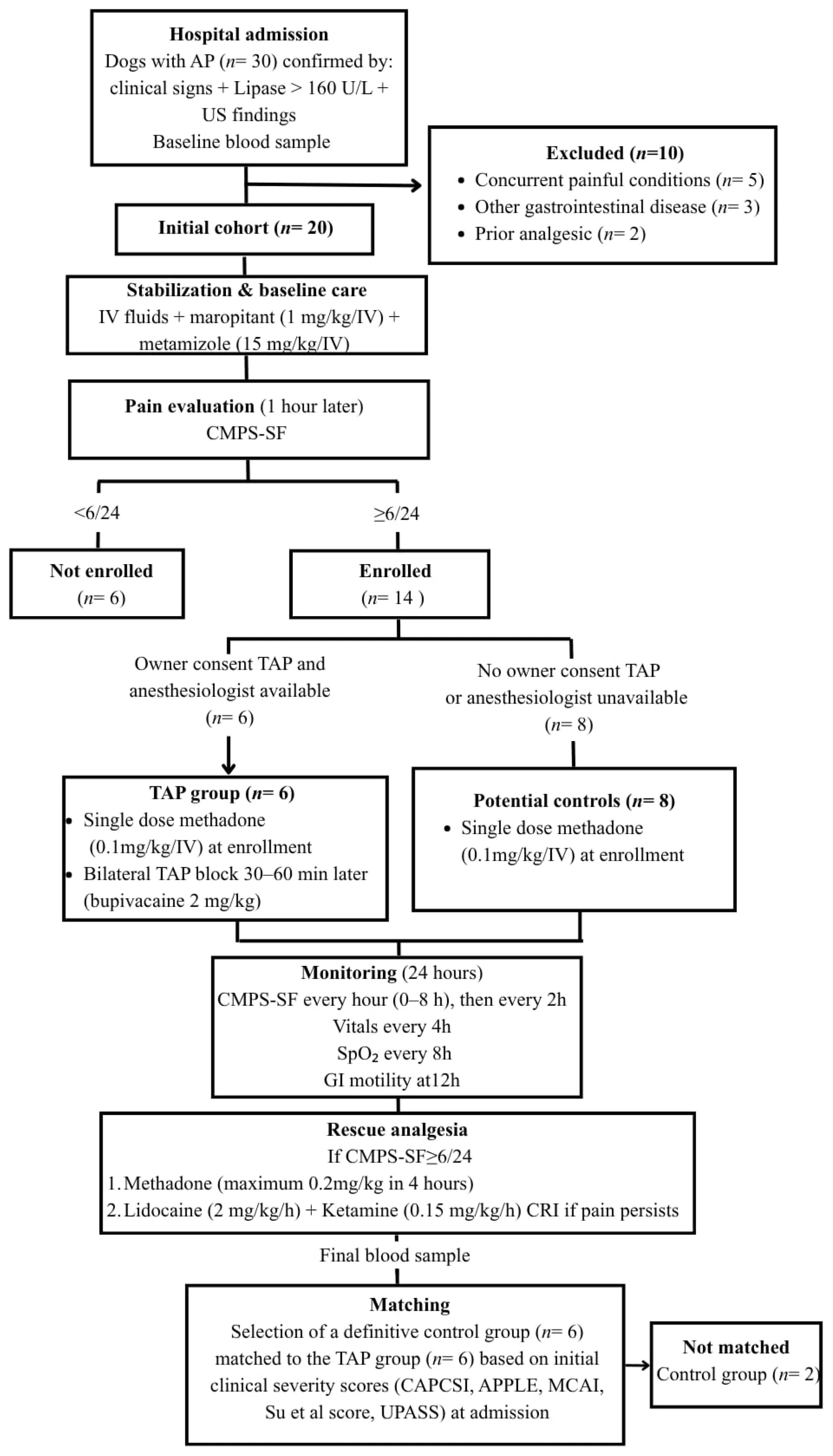
Study flow diagram detailing patient screening, group allocation, and clinical management. AP: acute pancreatitis; US: ultrasound; CMPS-SF: short form of the Glasgow Composite Measure Pain Scale [31]; CRI: constant rate infusion; CAPCSI: Canine Acute Pancreatitis Clinical Severity Index [1]; APPLE: Acute Patient Physiologic and Laboratory Evaluation [28]; MCAI: Modified Canine Activity Index [29]; UPASS: Ultrasound Pancreas-specific Scoring System [25].

Of the 8 dogs in the control pool, six controls were selected by 1:1 matching without replacement, minimizing the Euclidean distance across the five standardized admission severity scores (CAPCSI [1], APPLE [28], MCAI [29], UPASS [25], and Su et al. score [30]); the allocation minimizing the total distance was obtained by solving the corresponding linear assignment problem [32]. Matching was performed before any outcome variable was examined. The study flow is summarized in Figure 1.

### 2.4. Analgesic Interventions

Because a pain score ≥ 6/24 was the enrollment criterion, all enrolled dogs received methadone (Semfortan 10 mg/mL; Dechra Veterinary Products SLU, Barcelona, Spain; 0.1 mg/kg IV) at T0. This dose was identical in both groups but served a different purpose: in the control group it represented the first dose of opioid analgesia, whereas in the TAP group it was administered to prevent pain during performance of the block. Because it was common to both groups, this enrollment dose was not included in the count of rescue methadone doses. Metamizole and maropitant were maintained in both groups throughout hospitalization. The TAP block was thus evaluated as an adjunct within a multimodal protocol and not as a stand-alone analgesic technique.

- TAP group. The block was performed between 30 and 60 min after the enrollment dose of methadone. Alfaxalone (Alfaxan Multidosis 10 mg/mL; Zoetis Spain S.L., Madrid, Spain; 0.5–0.8 mg/kg, diluted 1:10 in sterile saline) was administered intravenously to effect to achieve adequate restraint. The injection sites were prepared with chlorhexidine and alcohol; the ventral abdomen had already been clipped in all dogs for the admission ultrasound examination. A bilateral, two-point ultrasound-guided TAP block was then performed, following the subcostal and preiliac approach described by Espadas-González et al. [20], using a linear 11.7 MHz transducer attached to a GE Logiq S7 Expert ultrasound unit (GE Healthcare, Milwaukee, WI, USA) and a 22-gauge echogenic needle (LOCOCARE; Ecuphar Veterinaria S.L.U., Barcelona, Spain), the length being chosen according to the size and body condition of each dog. At each of the four injection points, bupivacaine 0.5% (B. Braun, Rubí, Spain) was administered at 0.5 mg/kg, diluted with 0.9% sodium chloride (B. Braun Vet Care, Rubí, Spain) to a final volume of 0.3 mL/kg per point, for a total bupivacaine dose of 2 mg/kg. All blocks were performed by the same experienced anesthesiologist, in a separate room housing the ultrasound equipment and away from the hospitalization area.
- Control group. No block was performed.

### 2.5. Monitoring, Pain Assessment and Rescue Analgesia

**Figure 2:**
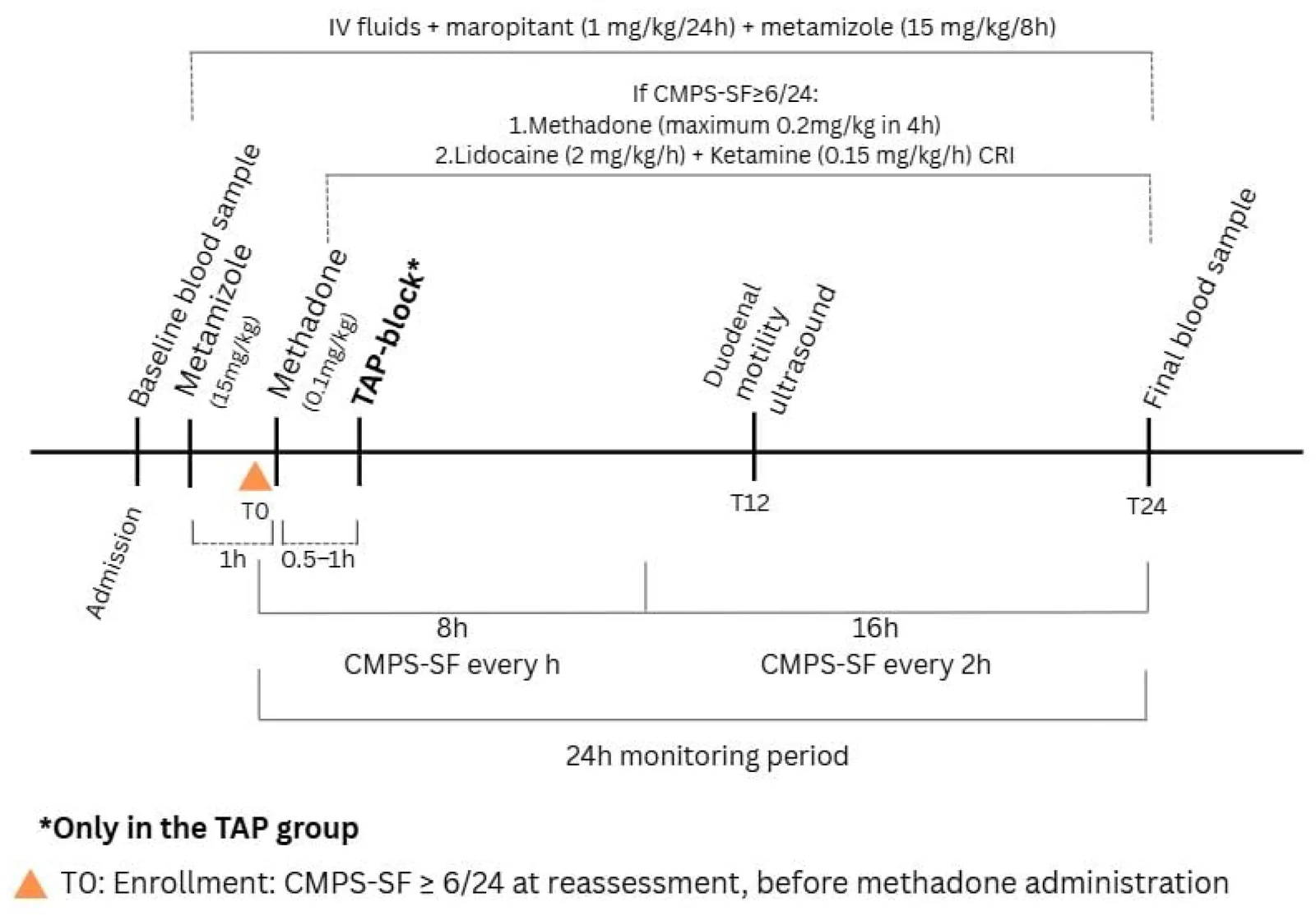
Timeline of the study protocol. T0 was defined as the first CMPS-SF assessment performed after metamizole, at which dogs scoring ≥ 6/24 (orange triangle) were enrolled and immediately received methadone. In the TAP group only (asterisk), a bilateral two-point block (bupivacaine 2 mg/kg) was performed 30–60 min later. Blood samples for C-reactive protein were obtained at admission (baseline sample) and on completion of the monitoring period (final sample). Rescue analgesia was identical in both groups.

Monitoring was standardized in both groups during the 24 h following T0; in the TAP group this window therefore included the 30 to 60 min preceding the block. Pain was scored with the CMPS-SF hourly during the first 8 h and every 2 h during the remaining 16 h, by experienced veterinary staff who did not participate in the blocks and were blinded to each dog’s group allocation. Vital signs, comprising heart rate, respiratory rate, rectal temperature, mucous membrane colour, and systolic and mean arterial blood pressure measured by oscillometry (Vet20; SunTech Medical Inc., Morrisville, NC, USA), were recorded every 4 h, peripheral oxygen saturation (SpO_2_) was measured by pulse oximetry (LifeVet PT; Eickemeyer Medizintechnik für Tierärzte KG, Tuttlingen, Germany) every 8 h, and gastrointestinal motility was assessed by transabdominal ultrasonography at T12 using the same ultrasound unit. Blood samples for CRP measurement were obtained at admission (baseline sample) and on completion of the monitoring period (final sample). The study timeline is summarized in Figure 2.

The same rescue protocol was applied in both groups: methadone (0.1 mg/kg IV) whenever the CMPS-SF score was ≥6/24, repeated if necessary within the same 4-h period; if the score remained ≥6/24 after a cumulative dose of 0.2 mg/kg/IV within a 4-h period, a CRI of lidocaine (Lidocaína B. Braun 20 mg/mL, B. Braun Medical S.A., Rubí, Spain; 2 mg/kg/h) and ketamine (Ketamidor 100 mg/mL; VetViva Richter GmbH, Wels, Austria; 0.15 mg/kg/h) was started.

No further medications were added until 24 h had elapsed. Thereafter, analgesia was tailored to each patient according to clinical reassessment.

### 2.6. Outcome Measures and Clinical Endpoints

To evaluate the efficacy of the analgesic protocols and the clinical course of the patients, the following outcomes were recorded:

- Opioid consumption, defined as the number of methadone doses administered during the 24 h after T0, excluding the enrollment dose.
- Pain during the first 24 h after T0, assessed using the CMPS-SF and summarized for each dog as the area under the score–time curve (AUC_0–24_).
- Time to rescue methadone administration, measured from T0; dogs not requiring rescue methadone during the 24-h observation period were censored at 24 h.
- Length of hospitalization (days) and survival to discharge.
- Time to first voluntary food intake (h), measured from admission and analyzed in dogs that ate during hospitalization; the proportion of dogs that ate voluntarily was also recorded.
- Need for a second-line antiemetic therapy (metoclopramide CRI) due to the persistence of emesis despite maropitant.
- Duodenal motility at T12 defined as <4 contractions/min [33] on ultrasonography.
- Change in CRP concentration (ΔCRP), calculated as the value in the final sample minus the value in the baseline sample.
- Complications related to the block

### 2.7. Statistical Analysis

Statistical analysis was performed using SPSS Statistics 29.0 (IBM Corp., Armonk, NY, USA). Data normality was assessed with the Shapiro-Wilk test. Due to the small sample size and non-normal distribution, non-parametric methods were used. The Mann-Whitney U test was used for inter-group comparisons and to evaluate clinical outcomes, and Fisher’s exact test was used for categorical variables. The CMPS-SF area under the curve from 0 to 24 h (AUC_0–24_) was calculated for each dog using the trapezoidal rule and compared between groups using the Mann–Whitney U test. Time to first rescue methadone administration was analysed using Kaplan–Meier analysis, with dogs not requiring rescue analgesia censored at 24 h; groups were compared using the log-rank (Mantel–Cox) test. Statistical significance was set at *p* < 0.05.

## 3. Results

### 3.1. Baseline Characteristics

Of the 30 dogs screened, 10 were excluded at admission because of a concurrent painful condition (*n* = 5), another gastrointestinal disease (*n* = 3), or previous analgesic treatment (*n* = 2). Of the remaining 20 dogs, 6 had a pain score < 6/24 one hour after metamizole and were not enrolled. Of the 14 enrolled dogs, 6 received the TAP block and 8 formed the control pool, of which 6 were selected by matching (Figure 1). The final study population therefore consisted of 12 dogs.

All dogs had shown clinical signs for less than 3 days before admission. Possible triggers were dietary indiscretion (*n* = 5) and habitual consumption of high-fat human food (*n* = 2); no trigger was identified in the remaining five dogs. The population included five mixed-breed and seven purebred dogs. The TAP group comprised two mixed-breed dogs, two Ratonero Bodeguero Andaluz, one Chihuahua and one English Pointer; the control group comprised three mixed-breed dogs, one Labrador Retriever and two Portuguese Podencos.

Baseline characteristics of the matched groups are shown in Table 1. Age, body weight, and body condition score did not differ significantly between groups, nor did the severity scores or CRP concentration at admission. Sex distribution was also comparable (male/female, 1/5 in the TAP group vs. 4/2 in the control group).

### 3.2. Clinical Outcomes and Efficacy

The clinical outcomes and analgesic requirements for both groups during hospitalization are summarized in Table 2.

A statistically significant reduction in opioid consumption was observed in the TAP group compared with the control group during the first 24 h after T0. Five of the six dogs (83.3%) that received the locoregional block remained opioid-free throughout this period, and the remaining dog received five rescue doses of methadone from 8 h onwards; this was also the dog in the TAP group that did not survive to discharge. In contrast, all control dogs required repeated methadone administration.

Beyond the monitoring period, three of the five opioid-free dogs in the TAP group remained comfortable on the baseline protocol until discharge, one required additional opioid analgesia, and one received a second TAP block because of recurrence of pain.

The CMPS-SF AUC_0–24_ was significantly lower in the TAP group than in the control group [68 (51.5–134) vs. 116.5 (106–135); *p* = 0.037], indicating lower cumulative observed pain scores during the first 24 h.

Time to first rescue methadone differed significantly between groups (log-rank test, χ^2^ = 10.735, *p* = 0.001). All six control dogs required rescue methadone within the first 6 h, with a median time to first rescue of 4 h (95% CI, 1.6–6.4). In the TAP group only one dog required rescue methadone at 8 h.

Regarding nutritional recovery, the time to first voluntary food intake was significantly shorter in the TAP group compared to the control group. The proportion of dogs that did not consume food voluntarily during their entire hospitalization was identical in both cohorts.

No statistically significant differences were observed between groups regarding the length of hospitalization (*p* = 0.254), the incidence of duodenal hypomotility at T12 (*p* = 0.242), the requirement for second-line antiemetic therapy (*p* = 0.182), or the systemic inflammatory response (ΔCRP, *p* = 0.132). Nevertheless, median ΔCRP was positive in the control group (2.75 mg/L) and negative in the TAP group (−0.90 mg/L), suggesting different patterns of change from baseline, although the difference was not significant (*p* = 0.132).

**Figure 3:**
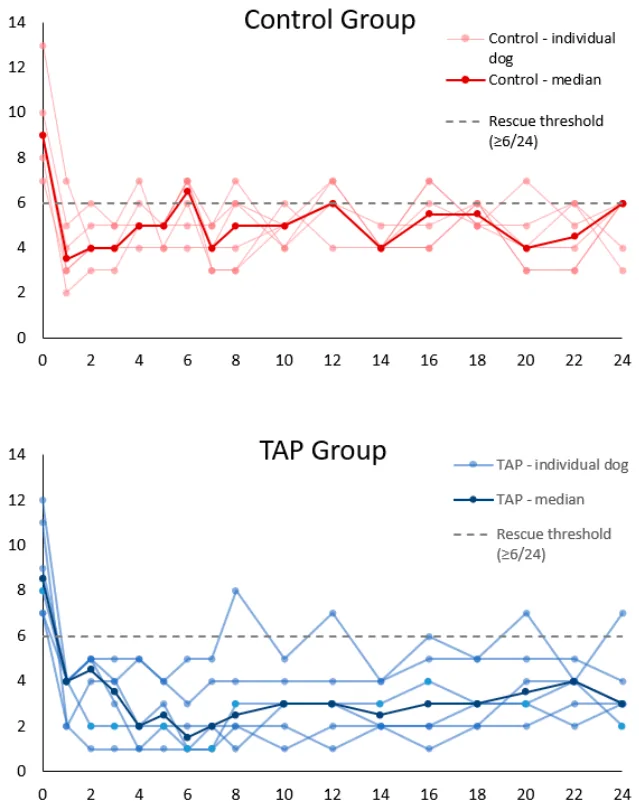
Individual CMPS-SF scores during the 24 h following T0 in the TAP group and the control group. Thin lines represent individual dogs (*n* = 6 per group) and thick lines the group median. The dashed line marks the rescue threshold (≥6/24) at which methadone was administered. Scores were recorded hourly during the first 8 h and every 2 h thereafter.

Individual CMPS-SF trajectories over the 24-h monitoring period are shown in Figure 3. In the control group, scores fluctuated around the rescue threshold throughout the period, repeatedly returning to ≥6/24 after each methadone dose, whereas in the TAP group scores fell after the block and remained below the threshold in most dogs.

Throughout hospitalization, SpO_2_ remained stable in all patients, with no values below 98%.

The survival-to-discharge rate was identical in both the TAP and control groups. Finally, no systemic adverse effects or major complications associated with the TAP block were observed. Mild bleeding at the injection site occurred in two dogs, which resolved rapidly with manual pressure.

## 4. Discussion

The preliminary findings of the present study suggest that incorporating the TAP block into a multimodal analgesic protocol may provide effective analgesia and reduce the consumption of systemic opioids in dogs with abdominal pain secondary to acute pancreatitis. Given the exploratory nature of this design and the limited sample size, these findings should be interpreted as an initial signal rather than definitive evidence of efficacy. The marked decrease in methadone requirements during the first 24 h after T0, achieved while cumulative pain scores remained lower in the TAP group over the same period, highlights the potential clinical value of this technique.

Pain during acute pancreatitis results from localized inflammation and the release of digestive enzymes, leading to an acute abdomen [34]. The TAP block addresses this condition by providing regional anesthesia to the abdominal wall, including the parietal peritoneum. Cadaveric studies demonstrate that injectate within the transversus abdominis fascia stains the segmental branches of spinal nerves T11 to L3 [12]. However, cadaveric models may not fully replicate in vivo anesthetic spread due to differences in muscle tone and fluid dynamics. Based on these models, distribution is predominantly central (T13–L2), while coverage at the cranial (T11–T12) and caudal (L3) extremes is inconsistent, potentially requiring supplementary analgesia [12].

Although the TAP block is a somatic technique and does not directly target pancreatic visceral afferents, our clinical results suggest effective analgesia, contrary to the view that significant visceral coverage would not be expected from this block [35]. A direct anatomical explanation may lie in the parietal peritoneum. In severe AP, the inflammatory process extends beyond the pancreas to the peripancreatic tissues and the adjacent parietal peritoneum, which, unlike the visceral peritoneum, receives somatic innervation from the ventral branches of the thoracolumbar spinal nerves, the same branches reached by injectate deposited in the transversus abdominis plane [12]. Desensitizing the abdominal wall and the inflamed parietal peritoneum therefore targets a substantial somatic component of pain in acute pancreatitis rather than merely its referred manifestation. This is consistent with the limited veterinary evidence available regarding the use of the TAP block in the management of abdominal pain in dogs [24]. Although direct extrapolation between species should be made cautiously, somatic and visceral afferents overlap in the dorsal horn, so that blocking thoracolumbar afferents may not only treat secondary somatic pain but also indirectly modulate visceral pain by attenuating central sensitization [36,37]. This mechanism may partially explain the opioid-sparing effect observed in our TAP cohort.

To date, veterinary literature evaluating the TAP block for medical abdominal pain remains limited. Freitag et al. [24] described its use in a small case series of three dogs presenting with acute abdomen of diverse etiologies. In that study, a continuous TAP infusion via a catheter was used, which limits a direct comparison with the single-injection protocol applied in the present study. The inclusion of a control group matched on five admission clinical severity scores was intended to reduce confounding and improve comparability between groups, although residual bias cannot be excluded.

In our study, the primary outcome—opioid consumption— was significantly lower in the TAP group compared to the control group (*p* = 0.015), indicating a marked opioid-sparing effect. Since methadone was administered in both groups only when the pain score reached the same threshold, the number of doses reflects how often pain returned: all control dogs required their first rescue dose within 6 h (median 4 h) and continued to require repeated doses throughout the 24-h period, whereas five of the six dogs in the TAP group did not require methadone beyond the enrollment dose. The methadone dose used here, 0.1 mg/kg IV, lies at the lower end of the range recommended for dogs in current pain management guidelines [10,11]; as the study evaluated an opioid-sparing strategy, analgesia was titrated to the pain score from this dose rather than started at a fixed higher dose. The reported terminal half-life of methadone after intravenous administration in dogs, approximately 4 h [38], underlies the 4-h interval and the 0.2 mg/kg cap applied before escalating to a lidocaine–ketamine infusion.

As a single injection of bupivacaine lasts hours rather than days, the benefit was necessarily time-limited: among those five dogs, two required further analgesia once the block had resolved —additional opioid in one case and a repeated TAP block in the other— whereas three remained comfortable without requiring further analgesic escalation and were maintained on the baseline protocol until discharge. This sustained comfort in half of the TAP cohort may reflect prevention of central sensitization or the natural improvement of the inflammatory process [19], which our design cannot distinguish.

Cumulative pain scores were also lower in the TAP group (AUC_0–24_ 68 vs. 116.5 score × h; *p* = 0.037). Because methadone was administered at the same threshold in both groups, pain scores were constrained within a similar range by design, so the lower AUC indicates that dogs in the TAP group remained further below that threshold rather than oscillating around it.

The individual trajectories (Figure 3) show two distinct patterns. In the control group, scores decreased after each methadone dose and returned to the intervention threshold within a few hours, so that the group median remained close to 6/24 for most of the monitoring period. In the TAP group, scores decreased during the first hours after the block and the group median remained between 2 and 4/24 from 4 h onwards, with individual values crossing the threshold only occasionally. Thus, the main difference between groups lay in the subsequent course of the pain scores rather than in their baseline values, with consistently lower scores over time in the TAP group, which is reflected in the lower AUC_0–24_.

Our results are in line with a recent prospective study in human patients with acute pancreatitis [18], which reported a reduction in total opioid consumption and a shorter hospitalization time. Furthermore, the study determined that pain reduction had an early onset and remained consistent throughout the 24-h monitoring period. This observation aligns with the clinical progression of our patients, in which five of the six dogs in the TAP group remained free of additional opioid requirements during the first 24 h. Similarly, in ten patients with acute pancreatitis and pain refractory to non-opioid analgesia, an inclusion criterion equivalent to ours, a bilateral TAP block provided almost immediate relief, although without a control group [19]. Before that study, evidence in human medicine was limited to isolated case reports and short series, mostly of chronic pancreatitis [36,39].

Regarding the length of hospitalization, the median stay was 1.5 days shorter in the TAP group [3 days (1–5) vs. 4.5 days (1–8)]. Although this difference did not reach statistical significance (*p* = 0.254), its magnitude may be clinically relevant if confirmed in adequately powered studies. A recent human study reported a significant reduction in hospitalization time with the TAP block [18], and our data point in the same direction without being able to establish it. Mortality was identical in both groups, with one fatal case in each cohort associated with severe systemic complications, which suggests that survival may be more closely related to disease severity and progression than to the analgesic protocol.

All dogs presented with anorexia upon admission, and a statistically significant difference was observed in favor of the TAP group (*p* = 0.02) in the time to first voluntary food intake. Among dogs that consumed food during hospitalization (four per group), the time to intake was shorter in the TAP cohort [12 h (8–16)] compared to the control group [48 h (24–72)]. This early resumption of feeding is clinically relevant, as the early enteral nutrition is considered a favorable factor for recovery in acute pancreatitis [5]. However, food intake in hospitalized dogs is multifactorial and may be influenced by behavioral and environmental factors, including stress and owner absence.

Regarding antiemetic requirements, none of the six dogs in the TAP group required second-line antiemetic therapy, compared with three of six controls (0% vs. 50%). This difference did not reach statistical significance (*p* = 0.182), which, with the present sample size, does not exclude a clinically meaningful effect. Opioids may exert both emetic and antiemetic effects in dogs depending on the dose, the route, and the particular drug [40], and nausea and vomiting requiring antiemetic rescue have been reported in dogs receiving repeated methadone [41]; a contribution of cumulative opioid exposure in our control group, which received a median of 4.5 doses over 24 h, cannot therefore be excluded. A possible analgesic contribution of maropitant must also be considered: as a selective NK-1 receptor antagonist, it blocks substance P, a neuropeptide involved in central pain processing and neurogenic inflammation [42], and experimental work in dogs has shown a reduction in anaesthetic requirement during noxious visceral stimulation [43] and intraoperative antinociceptive effects [44,45]. Clinical evidence nevertheless remains limited and inconsistent, with a systematic review finding no significant effect on pain [46], and the relevance of this mechanism to the pain of peritonitis has not been established. In any case, maropitant was administered at the same dose to all dogs, so this effect was controlled for in both groups. This finding cannot be conclusively attributed to the TAP block and may also reflect individual variability or disease progression.

The systemic use of opioids inhibits gastrointestinal transit by reducing acetylcholine release in enteric cells, altering neuronal excitability [47], and preventing coordinated peristalsis [48]. This iatrogenic dysmotility is associated with clinically relevant complications, including intolerance to enteral nutrition, aspiration pneumonia, bacterial overgrowth, and translocation [6,49,50,51]. It is important to consider that intra-abdominal diseases such as acute pancreatitis inherently reduce gastrointestinal motility [6]. Consequently, opioid administration in these patients may further exacerbate the risk of developing paralytic ileus. In this context, locoregional analgesia techniques may be highly beneficial by providing effective pain control while minimizing gastrointestinal adverse effects.

In the ultrasound assessment of gastrointestinal motility at T12, hypomotility (<4 contractions/minute) [33] was present in one of six dogs in the TAP group and in four of six controls, an absolute difference of 50 percentage points (16.7% vs. 66.7%). With six dogs per group, this difference did not reach statistical significance (*p* = 0.242), but an effect of this magnitude is clinically relevant and warrants evaluation in an adequately powered study. Although ultrasonography is a useful and non-invasive tool, its quantitative assessment is subject to variability and lacks well-defined reference ranges in diseased animals [6]. Additionally, gastrointestinal motility is influenced by physiological factors such as feeding status [52], and some healthy fasted dogs show no duodenal contractions [53]. Given that dogs in the TAP group resumed food intake earlier, it is difficult to determine whether the observed differences in motility were related to reduced opioid use or postprandial effects. Maropitant, given to all dogs, reduces gastric and jejunal but not significantly duodenal peristalsis [53].

The evolution of the inflammatory response was evaluated through changes in CRP levels between the baseline and final samples, as this biomarker is considered useful for monitoring inflammation in dogs [2,27,54]. CRP variation during the first 24 h followed a different pattern in each group: median ΔCRP was negative in the TAP group (−0.90 mg/L), indicating a decrease from baseline, and positive in the control group (2.75 mg/L), indicating an increase. However, the difference between groups was not statistically significant (*p* = 0.132). Consequently, these findings do not support a measurable effect of the TAP block on systemic inflammation, although they suggest that the procedure does not exacerbate the inflammatory response, consistent with previous studies indicating that the TAP block does not increase inflammatory markers in bitches undergoing laparoscopic ovariectomy [55].

Finally, all patients maintained stable SpO_2_ values (≥98%) and normal respiratory patterns during the first 24 h.

In summary, the current findings suggest that the ultrasound-guided TAP block may be a useful opioid-sparing adjuvant in the analgesic management of canine acute pancreatitis, evaluated as part of a multimodal protocol rather than as a stand-alone technique. The main limitations are the small sample size and single-center nature, limiting generalizability. Additional limitations include a non-randomized design with potential selection bias since allocation depended on anesthesiologist availability and owner consent; and restricted case inclusion due to requiring the same anesthesiologist and sonographer for all procedures. Blinding of the staff performing the pain assessments cannot be considered complete: dogs in the TAP group received alfaxalone for restraint, and residual sedation may have been perceptible at the earliest assessments. Several secondary outcomes differed by a magnitude that would be clinically relevant, and are reported here as signals to be tested in adequately powered studies rather than as established effects. Further multicenter studies with a larger sample size are needed to confirm these findings.

## 5. Conclusions

In conclusion, this preliminary study suggests that incorporating the ultrasound-guided TAP block into a multimodal analgesic protocol may reduce systemic opioid requirements in dogs with acute pancreatitis while maintaining lower pain scores during the first 24 h. No major block-related complications were observed in this small cohort and was associated with a shorter time to voluntary food intake. However, given the exploratory nature of this study and its limited sample size, these findings should be interpreted as preliminary. Further randomized multicenter studies are required to confirm these results and define the clinical role of this locoregional technique.

## Figures and Tables

**Table 1:** Baseline signalment and clinical severity indices in dogs with acute pancreatitis in the TAP and control groups.

	TAP Group (*n* = 6)	Control Group (*n* = 6)	*p*-Value
Age (years)	9 (2–16)	9.5 (1–15)	0.937
Body Weight (kg)	9.03 (3.4–28.5)	20.4 (7–23.7)	0.173
BCS	4 (2–5)	4 (2–5)	0.613
Sex (male/female)	1/5	4/2	0.242
CAPCSI	3 (2–4)	3 (3–5)	0.302
APPLE	30.5 (13–37)	24.5 (18–44)	0.936
MCAI	12 (9–16)	13 (10–15)	0.466
Su et al. [30] score	21 (11–63)	20.5 (11–42)	0.810
UPASS	4.5 (3–6)	4 (3–6)	0.614
CRP baseline sample (mg/L)	7.45 (0.9–29)	10.55 (1.5–24.6)	0.575

BCS: Body Condition Score; CAPCSI: Canine Acute Pancreatitis Clinical Severity Index [1]; APPLE: Acute Patient Physiologic and Laboratory Evaluation [28]; MCAI: Modified Canine Activity Index [29]; Sue et al. score: clinical severity scoring system described in reference [30]. UPASS: Ultrasound Pancreas-specific Scoring System [25]; CRP: C-reactive protein. Data are shown as median and min-max. Between-group comparisons were performed using the Mann–Whitney U test for continuous variables and Fisher’s exact test for categorical variables.

**Table 2:** Clinical outcomes in dogs with acute pancreatitis treated with TAP block or standard opioid-based analgesia.

Outcome	TAP Group (*n* = 6)	Control Group (*n* = 6)	*p*-Value
Methadone rescue doses (24 h)	0 (0–5)	4.5 (4–7)	0.015
Opioid-free during first 24 h	5/6 (83.3%)	0/6 (0%)	0.015
AUC_0–24_ CMPS-SF	68 (51.5–134)	116.5 (106–135)	0.037
Hours to first voluntary food intake *	12 (8–16)	48 (24–72)	0.02
Voluntary food intake	4/6 (66.7%)	4/6 (66.7%)	1
Length of hospitalization (days)	3 (1–5)	4.5 (1–8)	0.254
Second-line antiemetic required	0/6 (0%)	3/6 (50%)	0.182
Duodenal hypomotility at T12	1/6 (16.7%)	4/6 (66.7%)	0.242
CRP, final sample (mg/L)	6.35 (0.8–17.5)	16.35 (1.6–25.7)	0.150
ΔCRP (mg/L)	−0.90 (−13.4–0)	2.75 (−3.90–6.10)	0.132
Survival to discharge	5/6 (83.3%)	5/6 (83.3%)	1

AUC_0–24_ CMPS-SF: cumulative pain. Between-group comparisons were performed using the Mann–Whitney U test or Fisher’s exact test, as appropriate. * Calculated among dogs that consumed food voluntarily during hospitalization. ΔCRP: change in C-reactive protein concentration between the baseline and final samples. Reference interval for CRP: 0–0.9 mg/L.

## Data Availability

The clinical data generated and analyzed in this study belong to the participating veterinary hospital. Because of confidentiality and privacy policies regarding patient medical records, the datasets are not publicly available. Anonymised data may be provided by the corresponding author on reasonable request and with the approval of the data owner.

## Abbreviations

The following abbreviations are used in this manuscript:

AP	Acute Pancreatitis
APPLE	Acute Patient Physiologic and Laboratory Evaluation
AUC	Area Under the Curve
BCS	Body Condition Score
CAPCSI	Canine Acute Pancreatitis Clinical Severity Index
CMPS-SF	Short-form Glasgow Composite Measure Pain Scale
CRI	Constant Rate Infusion
CRP	C-reactive protein
MCAI	Modified Canine Activity Index
SpO_2_	Peripheral capillary oxygen saturation
TAP	Transversus Abdominis Plane
UEx	University of Extremadura
UPASS	Ultrasound Pancreas-specific Scoring System
VTH-UEx	Veterinary Teaching Hospital of the University of Extremadura
